# Belarus and drug-resistant tuberculosis

**DOI:** 10.2471/BLT.19.021219

**Published:** 2019-12-01

## Abstract

A comprehensive approach to delivering patient-centred treatment underpins Belarus’s progress against drug resistant tuberculosis. Andrey Shukshin and Gary Humphreys report.

From his office in the tuberculosis centre (National Scientific and Practical Centre for Pulmonology and Tuberculosis), just outside Minsk, Belarus, Director Gennady Hurevich has a front row view of the emerging pandemic of drug-resistant tuberculosis.

Drug-resistant tuberculosis is of concern worldwide. According to the World Health Organization’s (WHO) *Global tuberculosis report 2019*, almost half a million people have tuberculosis infections that are resistant to rifampicin, a mainstay of tuberculosis treatment since the 1970s. India has the largest share of drug resistant tuberculosis, with 27% of total global new cases.

Some 390 000 of the people with rifampicin resistant tuberculosis (78%) have infections that are also resistant to isoniazid (a first-line drug from the 1950s). These infections are defined as multidrug-resistant tuberculosis (MDR-TB).

Belarus is one of three countries in Eastern Europe – the others being the Russian Federation and Ukraine – which have the highest proportions of MDR-TB within their tuberculosis-infected populations.

According to WHO’s *Tuberculosis surveillance and monitoring in Europe 2019*, Belarus had the highest proportion of MDR-TB infections among previously untreated patients in 2017 at 37.2% (629/1690).

Hurevich has watched as once-effective drugs have stopped working, and treatment regimens have become more difficult, often involving injectable drugs.

“For patients with the multi-drug resistant infections, life is extremely challenging,” Hurevich says. “These are mostly poor people, often isolated because of their disease and mostly very afraid.”

When, in 2013, WHO issued a conditional recommendation for bedaquiline, the first new anti-tuberculosis drug in almost fifty years, Hurevich was keen to bring it to the patients in need.

The Belarus national tuberculosis programme began treatment with bedaquiline-containing regimens in June 2015. The programme followed WHO's interim policy guidance, including its criteria for eligible patients, procedures for informed consent and active pharmacovigilance for side effects.

“Of 197 patients treated, two died, and we observed sputum culture conversion (the point at which samples taken from a person infected with tuberculosis can no longer produce cultures) in 186 patients six months after commencement,” says Alena Skrahina, deputy director and head of research at the Minsk tuberculosis centre.

The results were striking given that 60% of the 197 patients had extensively drug resistant tuberculosis (XDR-TB), defined as infections resistant to rifampicin and isoniazid, at least one fluoroquinolone and at least one of the three injectable drugs: kanamycin, amikacin or capreomycin.

“One new drug alone [….] cannot make a difference unless it is delivered to the right people in the right way.”

Gennady Hurevich

Since then, several studies have confirmed bedaquiline’s efficacy. A cohort study undertaken in Belarus in 2018 involving 181 patients suffering from various forms of drug resistant tuberculosis, including XDR-TB, achieved a 93% cure rate. “That is comparable to outcomes observed in ordinary drug-susceptible TB treatment,” Skrahina says.

Despite these promising results, four years after introducing the drug, Hurevich is realistic about the challenges faced in tackling his country’s MDR-TB epidemic and gets annoyed when he hears the difficulties being minimized.

“There is no such thing as a silver bullet,” he says. “One new drug alone, no matter how effective, cannot make a difference unless it is delivered to the right people in the right way. It has required a comprehensive effort to achieve what we have achieved here.”

Much of that effort has gone into identifying people infected with drug-resistant tuberculosis, which requires bacteriological confirmation of tuberculosis and testing for drug resistance using rapid molecular tests, culture methods or sequencing technologies.

Belarus is making extensive use of the Xpert MTB/RIF rapid diagnostic test which allows for simultaneous diagnosis of tuberculosis and detection of rifampicin resistance. According to the *Global tuberculosis report 2019, *all Belarus tuberculosis testing sites are equipped to do these tests.

However, the national programme continues to rely on people coming forward to be tested rather than looking for them. This is a problem because many people with active tuberculosis do not experience symptoms in the early stages of the disease and are unlikely to seek care early.

“It is important to catch cases early to avoid delayed treatment and transmission,” says Dr Medea Gegia, a technical officer in the Global Tuberculosis Programme.

Working with WHO, The Global Fund to Fight AIDS, Tuberculosis and Malaria and Médecins Sans Frontières (MSF), Belarus is also working to remove obstacles to treatment, one of which is the high cost of the medicines used.

Johnson & Johnson, the patent-holder of bedaquiline, sells a six-month treatment course of bedaquiline for US$ 400 to countries buying through the Global Drug Facility, a tuberculosis drug and diagnostic procurement mechanism operating out of the United Nations Office for Project Services.

On the open market, bedaquiline sells for three times that amount, which puts it out of reach of many patients, including patients in Belarus. Bedaquiline is only one in a combination of expensive drugs.

“Patients receive an expensive cocktail of drugs, which few would have been able to afford without outside help,” Hurevich points out.

The Belarus Ministry of Health and MSF have also been providing patients with food parcels since 2015, and the Global Fund and MSF provide transport vouchers, as well as paying for video-observed treatment, including the internet connection required to transmit it.

Such support allows patients to continue treatment at home, minimizing the risk of further spread of drug resistant tuberculosis. According to Hurevich, some 1000 patients in Belarus are now using video-observed treatment.

Finally, considerable effort has gone into developing infrastructure and strengthening the health workforce to deliver appropriate care. MDR-TB care is complicated by the need to use multiple drugs in combination, and to monitor patients for adverse reactions.

“Implementation of the tuberculosis programme required a lot of trust-building.”

Dmitry Vetushko

Belarus’ MDR–TB specialists have also had to counter negative social media messaging surrounding bedaquiline. “A number of my patients refused to give their informed consent to [receive] bedaquiline due to the negative information posted on some Russian-language websites,” says Dr Dmitry Vetushko, head of the MDR–TB department at the Minsk tuberculosis centre. “Implementation of the tuberculosis programme required a lot of trust-building with data in hand.”

In support of capacity-building efforts, the WHO office in Belarus contracted 14 Belarus national specialists to ensure active tuberculosis drug safety monitoring and management, backed by the Global Fund to Fight AIDS, Tuberculosis and Malaria.

Since initiating its national programme, Belarus has scaled-up the treatment of drug-resistant tuberculosis, reaching almost all people with MDR-TB. Of 30 countries with a high burden of MDR-TB, only Belarus and Kazakhstan have reached this level of coverage.

According to Dr Viatcheslav Grankov, a communicable diseases expert at the WHO Country Office in Belarus, all patients with MDR–TB are being treated in line with WHO’s 2019 guidelines.

WHO estimates that 20 of the 30 high MDR-TB burden countries have coverage below 50%, while a study published in the January 2018 issue of *The International Journal of Tuberculosis and Lung Disease* found that only 15.7% of the 69 213 people in 32 countries estimated to need bedaquiline or delaminid were reported to have received them outside of clinical trials or under compassionate use protocols during the study period of mid-2015 to mid-2017.

Five years since WHO’s End TB Strategy set out three main ‘pillars’ for the elimination of this disease, the third of which was intensified research and innovation to develop new medicines vaccines and diagnostics, considerable progress has been made.

According to WHO’s Global Observatory on Health R&D, there are currently 7 anti-tuberculosis products in Phase III trials, including three antibiotics and four vaccines. In addition, the candidate vaccine M72/AS01E was found to be significantly protective against tuberculosis disease in a Phase IIb trial in individuals with evidence of latent tuberculosis infection. If the findings are confirmed, the vaccine could transform tuberculosis prevention and control.

However promising, any new products that eventually emerge will need to be rolled out in the context of comprehensive efforts, such as those being made in Belarus. Moreover, those efforts will need to be underpinned by the broader socioeconomic reforms required to tackle this disease. Poverty reduction strategies and expanding social protection must therefore be part of any truly comprehensive response.

**Figure:**
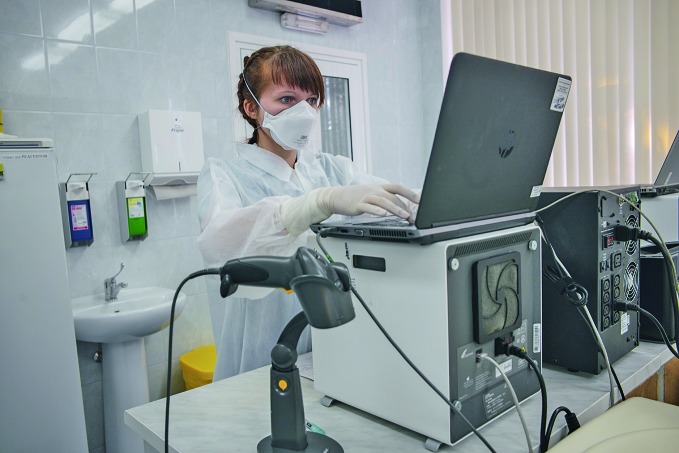
Laboratory at the National Scientific and Practical Centre for Pulmonology and Tuberculosis, Belarus, Minsk.

Viviane Dalles/ Médecins Sans Frontières 

**Figure:**
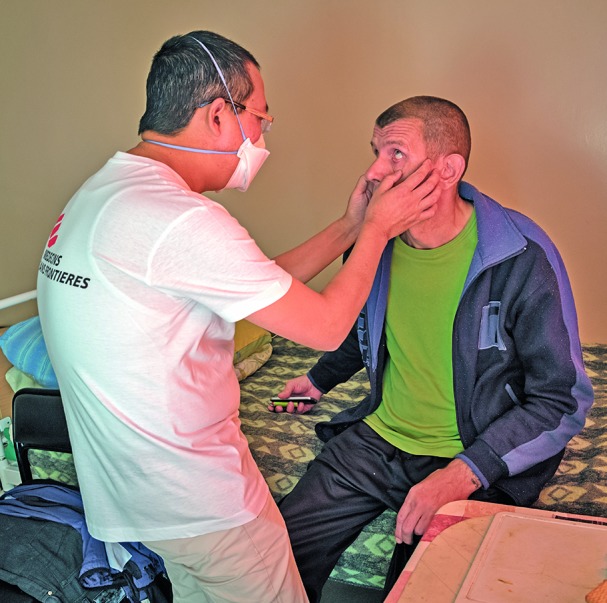
Patient Consultation at the National Scientific and Practical Centre for Pulmonology and Tuberculosis, Belarus, Minsk.

Viviane Dalles/ Médecins Sans Frontières 

